# *In situ* Nuclear Matrix preparation in *Drosophila melanogaster* enabling genetic analysis of the nuclear architecture

**DOI:** 10.1016/j.xpro.2022.101394

**Published:** 2022-05-13

**Authors:** Rashmi U. Pathak, Ashish Bihani, Rahul Sureka, Rakesh K. Mishra

**Affiliations:** 1 Centre for Cellular and Molecular Biology, Uppal Road, Hyderabad, Telangana 500 007, India; 2 Tata Institute for Genetics and Society, Bangalore, India

**Keywords:** Cell Biology, Microscopy, Model Organisms, Structural Biology

## Abstract

Nuclear Matrix (NuMat) is a biochemically defined entity that provides us with a snapshot of the features of the nuclear architecture. Here, we present a protocol to isolate and visualize NuMat *in situ* in the intact embryo or tissues of *Drosophila melanogaster* and its applications. We remove the chromatin to reveal underlying nuclear architectural components in organismal context. This protocol couples the power of *Drosophila* genetics with cell biological observation of the nuclear architecture.

For complete details on the use and execution of this protocol, please refer to Pathak et al. (2022), Sureka et al. (2018), and Pathak et al. (2013).

## Highlights

• Isolate and visualize NuMat *in situ* in embryos or tissues of *Drosophila melanogaster*
• Use in conjunction with *Drosophila* genetics to enable analysis of nuclear architecture
• Biochemically isolate components of nuclear architecture observable through microscopy

## Abstract (editor highlights)

Publisher's note: Undertaking any experimental protocol requires adherence to local institutional guidelines for laboratory safety and ethics.

## Graphical abstract

**Figure:**
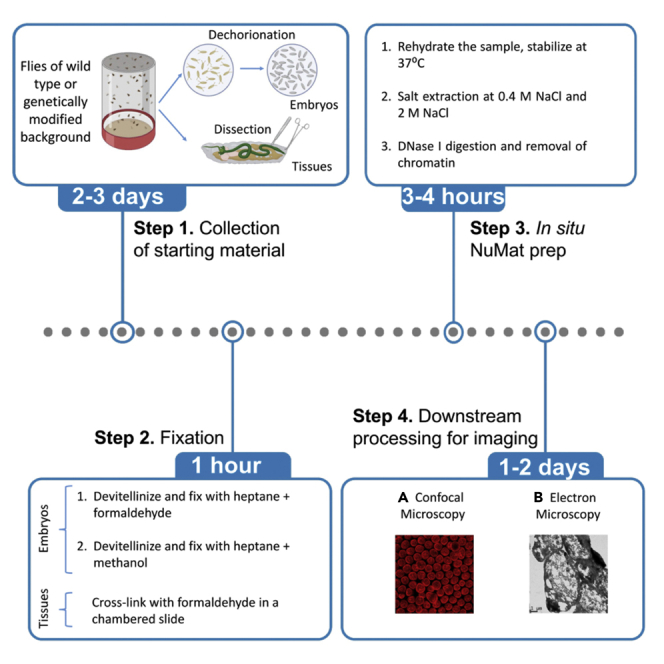

## Before you begin

This protocol is for the isolation of *in situ* NuMat and its visualization by transmission electron microscopy (TEM) and immuno-fluorescence confocal microscopy. However, the protocol can also be used to visualize DNA and RNA associated with nuclear architecture. In such a case, the isolated NuMat should be probed for target DNA/RNA by florescence *in situ* hybridization followed by confocal microscopy.

### Institutional permissions

Before you start isolation of *in situ* NuMat, you need to have permission to perform animal experiments. For us, all animals, in this case *Drosophila melanogaster*, were used as per the bio-safety and animal ethics procedure of our organization.

### General preparations

#### Reagent preparations

**Timing: 2–3 h**

1. Prepare grape-juice agar plates for embryo collection.
2. Prepare all the stock solutions.
3. Sterilize stock solutions by autoclaving.

#### Collection of *Drosophila* embryos

**Timing: 2–3 days**

**Figure 1:**
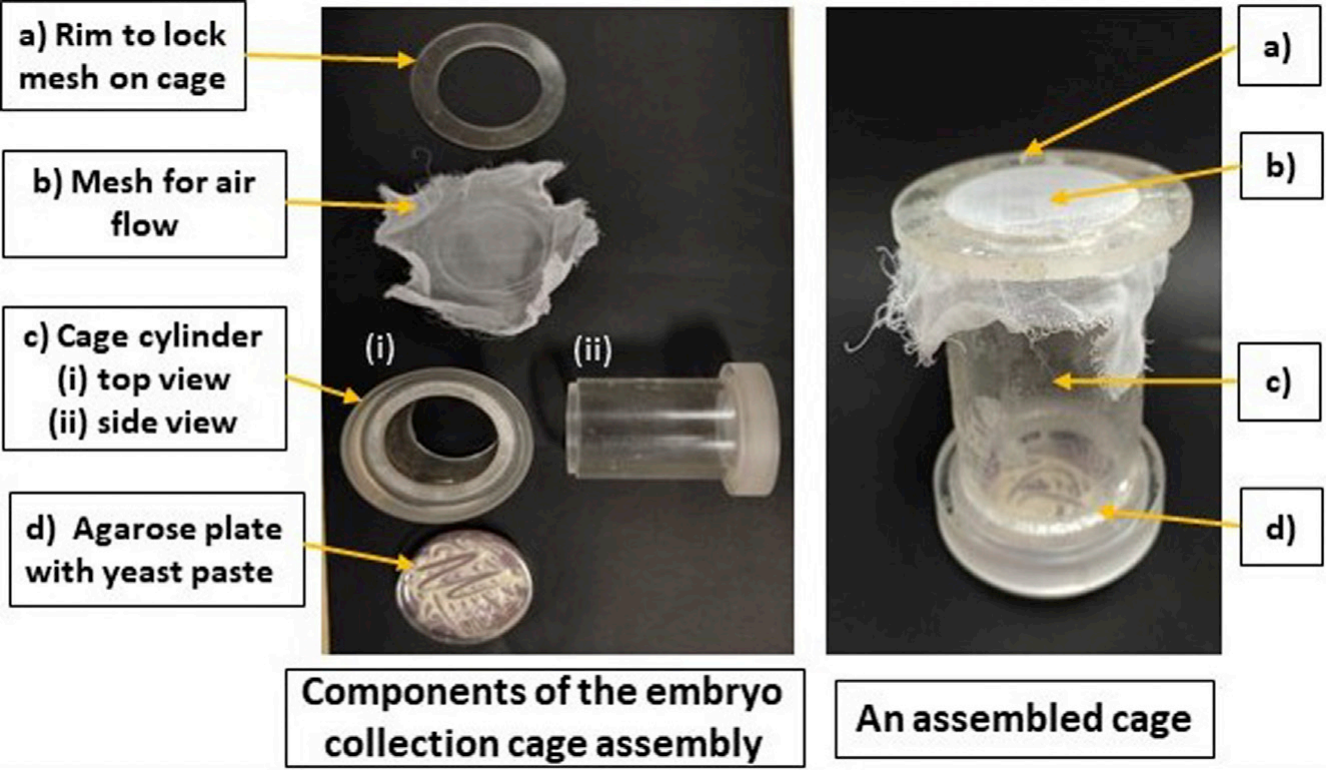
An embryo collection cage setup
6. Assemble the cage as shown in the figure and place it with the grape juice-agar plate side down at 25°C in an incubator with 12 h:12 h light:dark cycle for 24 h. This is to acclimatize the flies to the cage environment and maximize egg lay.
7. Check the surface of agar plates for eggs. If there are too few eggs, flies may require longer acclimatization period.
8. For embryo collection for *in situ* NuMat preparation, place a fresh plate on the cage. Smear a small amount of yeast paste onto the center of the plate. This addition attracts flies and enhances egg laying.
9. Discard the plate after 1 h, and replace it with a fresh one. As flies can with-hold the eggs before laying, this step is important to ensure that the embryos are synchronized and of a particular developmental stage.
10. Gently dislodge the eggs from agar surface with the help of a paint brush. Most eggs will be found toward the edge of the plate. Collect the eggs in 1× PBS in an Eppendorf tube and proceed with the step-by-step protocol as detailed below.

***Note:*** The cage used by us was fabricated in the institutional workshop, but equivalent assemblies are available for purchase.

4. Collect 2–10 days old male/female flies and put them in embryo collection cage at a density of 50 males and 100 females in each cage.
5. Set up a typical embryo collection cage as shown in Figure 1A.

## Key resources table

**Table:** 

REAGENT or RESOURCE	SOURCE	IDENTIFIER
**Antibodies**		

Lamin Dm0 mouse monoclonal (1:200)	DSHB, Univ of Iowa	ADL67.10
Histone H3 rabbit polyclonal (1:500)	Abcam	ab18521
Polycomb rabbit polyclonal (1:100)	Santa Cruz Antibodies	Sc-25762
Anti-Rabbit IgG, Alexa Fluor 488 (1:500)	Thermo Fisher scientific	A-11008
Anti-Mouse IgG, Alexa Fluor 488 (1:500)	Thermo Fisher scientific	A-21202
Anti-Rabbit IgG, Alexa Fluor 594 (1:500)	Jackson ImmunoResearch	711-585-152
Anti-Mouse IgG, Alexa Fluor 594 (1:500)	Jackson ImmunoResearch	711-585-150

**Chemicals, peptides, and recombinant proteins**		

DAPI	Sigma-Aldrich	Cat#D8417
DGD	Electron Microscopy Sciences	Cat#13255
DNase I	Sigma-Aldrich	Cat#D4527
EDTA	Sigma-Aldrich	Cat#E9884
Ethanol EM grade	Electron Microscopy Sciences	Cat#15055
Formaldehyde	Sigma-Aldrich	Cat#F8775
Glutaraldehyde EM grade	Sigma-Aldrich	Cat#G5882
HEPES	Sigma-Aldrich	Cat#H3375
Heptane	Merck	Cat#34873
Isopropanol	Sigma-Aldrich	Cat#I9516
KCl	Sigma-Aldrich	Cat#P9541
KH_2_PO_4_	Sigma-Aldrich	Cat#P5655
Na_2_HPO_4_	Sigma-Aldrich	Cat#S3264
Methanol	Merck	Cat#34860
NaCl	Sigma-Aldrich	Cat#S3041
N-butyl alcohol	Electron Microscopy Sciences	Cat#11920
Mounting media Vectashield Antifade	Vector Laboratories	Cat#H-1200-10
OsO_4_	Electron Microscopy Sciences	Cat#19192
PIPES	Sigma-Aldrich	Cat#P1851
PMSF	Sigma-Aldrich	Cat#P7626
Sodium Cacodylate	Electron Microscopy Sciences	Cat#11654
Sodium Hypochlorite solution	Fisher Scientific	Cat#27908
Sucrose	Sigma-Aldrich	Cat#S9378
Trizma	Sigma-Aldrich	Cat#T6066
Triton-X-100	Sigma-Aldrich	Cat#T8787

**Experimental models: Organism/strains**		

*Drosophila melanogaster*, Canton-S strain	Laboratory grown	N/A

**Software and algorithms**		

LAS X software	Leica Microsystems	Version 3.1.1.15751 (download link)

**Other**		

Chemical fume hood	Locally fabricated	N/A
Chambered slide (2 chamber)	Nalgene Nunc	Cat#155379
Confocal microscope	Leica Microsystems	SP8 X
Coverslips	Fisherbrand	Cat#12-544B
Embryo collection cages	Locally fabricated	N/A
Fine forceps (bent)	Fine Science Tools	Cat#11274-20
Fine forceps (straight)	Fine Science Tools	Cat#11254-20
Formvar coated copper grids	Electron Microscopy Sciences	Cat#CF400-CU
Glass for knives	Electron Microscopy Sciences	Cat#7890
Glass slides	Fisherbrand	Cat#12-550-400
Heating block	Eppendorf	Thermomixer Comfort
37°C and 60°C incubator	Sanyo	MIR-262
Stereomicroscope	Carl Zeiss	Stemi 2000
Loop and Handle	Electron Microscopy Sciences	Cat#70944
Dissection Needle	Locally purchased	N/A
Paint brush	Locally purchased	N/A
pH meter	Thermo Scientific	Eutech pH700
Platform shaker	Tarsons	Rockymax Cat#4080
Rotator	Tarsons	Rotospin Cat#3070
Transmission Electron Microscope	JEOL	JEM-2100
Ultramicrotome	Leica	EM UC7
Glass cavity block	Abgil	ABG20032

***Note:*** 1. Small equipment like p10, p20, p200 and p1000 pipettes and appropriate pipette tips and 1.5 mL Eppendorf tubes are also required. 2. Embryo collection cages are available for purchase at https://flystuff.com.

## Materials and equipment

**Table:** Required stock solutions

Reagent	Stock concentration	Amount
EDTA	0.5 M	10 mL
HEPES pH 7.5	1 M	10 mL
Tris-Cl pH 7.5	1 M	10 mL
KCl	3 M	10 mL
NaCl	5 M	10 mL
MgCl_2_	1 M	10 mL
PMSF	0.1 M	5 mL
Triton-X-100	10%	10 mL

All the stock solutions can be stored at 25°C for 3 months.

**Table:** Preparation of 10× PBS

Reagent	Final concentration	Amount
NaCl	1.37 M	80 gm
KCl	27 mM	2 gm
Na_2_HPO_4_	100 mM	17.8 gm
KH_2_PO_4_	18 mM	2.4 gm
Deionized H_2_O	N/A	800 mL

Adjust pH to 7.4 with 0.1 N HCl if needed.

Adjust final volume to 1,000 mL. Store at 25°C for 3 months.

**Table:** Preparation of 1× PBT

Reagent	Final concentration	Amount
10× PBS	1×	10 mL
10% Triton-X-100	0.5% (v/v)	5 mL

Adjust final volume to 100 mL with deionized water. Store at 25°C.

**Table:** Preparation of fixation buffer

Reagent	Final concentration	Amount
10× PBS	1×	1 mL
37% formaldehyde	4% (v/v)	1.08 mL

Adjust final volume to 10 mL with deionized water. Prepare fresh before every use. Store at 25°C for the day.

**Table:** Preparation of DNase I

Reagent	Final concentration	Amount
1 M Tris-Cl pH 7.5	10 mM	10 μL
DNase I	10 mg/mL	10 mg

Adjust final volume to 1 mL with deionized water.

Store in aliquots in −30°C up to 1 year.

**Table:** Preparation of Digestion buffer

Reagent	Final concentration	Amount
1 M Tris-Cl pH 7.5	10 mM	10 μL
3 M KCl	20 mM	6.6 μL
5 M NaCl	70 mM	14 μL
1 M MgCl_2_	10 mM	10 μL
10% Triton-X-100	0.5%	50 μL
0.1 M PMSF	1 mM	10 μL

Adjust final volume to 1 mL with deionized water. Prepare fresh before every use. Store at 25°C for the day.

**Table:** Preparation of Extraction buffer I (containing 0.4 M NaCl)

Reagent	Final concentration	Amount
1 M HEPES pH 7.4	5 mM	5 μL
3 M KCl	2 mM	0.6 μL
0.5 M EDTA	2 mM	4 μL
5 M NaCl	0.4 M	80 μL
10% Triton-X-100	0.5%	50 μL
0.1 M PMSF	1 mM	10 μL

Adjust final volume to 1 mL with deionized water. Prepare fresh before every use. Store at 25°C for the day.

**Table:** Preparation of Extraction buffer II (containing 2 M NaCl)

Reagent	Final concentration	Amount
1 M HEPES pH 7.4	5 mM	5 μL
3 M KCl	2 mM	0.6 μL
0.5 M EDTA	2 mM	4 μL
5 M NaCl	2 M	400 μL
10% Triton-X-100	0.5%	50 μL
0.1 M PMSF	1 mM	10 μL

Adjust final volume to 1 mL with deionized water. Prepare fresh before every use. Store at 25°C for the day.

**Table:** Preparation of Grape-juice Agar plates

Reagent	Final concentration	Amount
Sugar	3% (w/v)	30 gm
Yeast	1.7% (w/v)	17 gm
Agar	2.5% (w/v)	25 gm
Methyl benzoic acid	0.025% (w/v)	0.25 gm
Propionic acid	0.5% (v/v)	5 mL
Orthophosphoric acid	0.1% (v/v)	1 mL
Grape juice	33% (v/v)	333 mL

Adjust final volume to 1 liter. Heat in a microwave oven to dissolve. Pour into plates when cooled to ∼50°C. Store the plates at 4°C for a week.

**CRITICAL:** Formaldehyde is toxic. It is a carcinogen as well as corrosive. Always use the stock bottle in chemical fume hood and handle with extreme caution.

***Note:*** 1. Triton-X-100 and buffers containing it are not autoclaved. 2. PMSF is added to the buffers just before use.

## Step-by-step method details

### Dechorionation, fixation, and devitellinization of embryos

**Timing: 1 h**

The first step of dechorionation of embryos is critical for the success of rest of the protocol since chorion of the embryo forms a barrier and would inhibit all access to reagents in later steps. Optimal fixation and devitellinization ensures proper visualization of architectural components. Problem 1.

**Figure 2:**
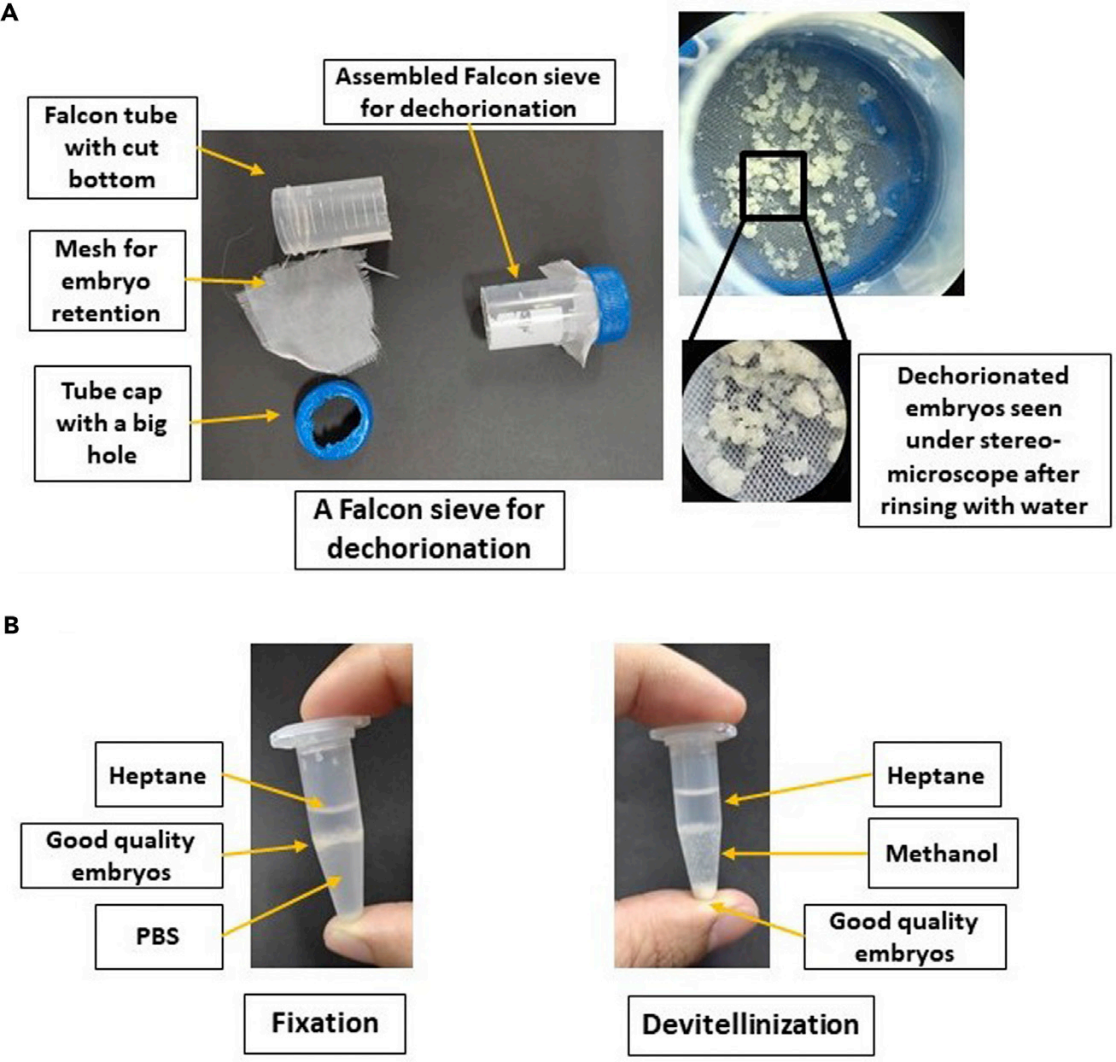
Dechorionation, fixation, and devitellinization of embryos setup (A) Dechorionated embryos collected and washed thoroughly in a home-made falcon sieve. (B) Fixed embryos form a ring at the Heptane-PBS interphase. Devitellinized embryos settle down promptly after addition of ice-cold methanol.
  d. Wash the embryos thoroughly with running tap water straining through the sieve mesh, till smell of bleach is no longer detectable.

1. Dechorionation:
  a. Collect 50–100 embryos in water in a small beaker with the help of a brush.
  b. Add bleach (Sodium hypochlorite) to the water to a final concentration of 50% (v/v) and treat the embryos for 2–3 min to remove the chorion. The complete removal of the chorion can be assessed by observing under the microscope.
  c. Collect the embryos in a home-made sieve made by cutting 50 mL Falcon tube (Figure 2A).

**CRITICAL:** Sodium hypochlorite is toxic and can de-stain clothes. While handling wear gloves and lab coat.

***Note:*** Bleach should be freshly diluted before use and not stored.

It is very important to remove residual bleach by rinsing thoroughly with tap water since bleach is a strong oxidizing agent and may interfere with the steps that follow. Problem 2.

2. Fixation and devitellinization:
  a. Rinse embryos twice with 1× PBS. 1× PBS can be prepared and stored at 4°C for one month.
  b. Collect embryos in a 1.5 mL Eppendorf tube and keep on ice for 10 min to chill. Size of tube and volume of fixative should be decided according to the number of embryos processed. For about 50–100 embryos, a 1.5 mL Eppendorf tube can be used. For higher numbers (>500 to 0.5 gm), a 15 mL screw cap polypropylene tube can be used.
  c. Fill the tube up to 80% with fixation buffer:heptane mix (1:1, v/v), to avoid loss of embryos due to sticking on the walls. The protocol detailed here is for ∼100 embryos that can be processed in a 1.5 mL Eppendorf tube.
  d. To fix, submerge embryos in 0.6 mL of fixation buffer (1× PBS containing 4% formaldehyde, prepare fresh just before use and do not store) and add equal volume of heptane (0.6 mL).
  e. Mix by vigorous shaking and then incubate the tube on a rotating wheel at 100 rpm at 25°C for 20 min. The NuMat isolation can also be done without fixation and for this formaldehyde should not be added to the devitellinization mix.
  f. Let the tube stand on ice for 2 min to allow separation of the aqueous and organic phases.
  g. Gently aspirate and discard the lower aqueous phase without sucking any embryos in the process. The good quality fixed embryos form a ring at the junction of aqueous phase and heptane (Figure 2B).
  h. To devitellinized the embryos, add methanol (pre-chilled at −20°C, volume equal to heptane). Shake vigorously and let devitellinized embryos settle at the bottom. Gently aspirate and discard supernatant.
  i. Wash these embryos twice, in quick succession, by adding 1 mL pre-chilled methanol, allowing the embryos to settle down and then discarding the supernatant by gentle aspiration.
  j. In all the steps of washing embryos, give time for embryos to settle down completely and then pipette out the solution from the tubes to prevent the loss of sample in the procedure. Fine tip should be used to aspirate solutions since it prevents loss of embryos.
  k. Only the good quality embryos that settle down to the bottom should be used for NuMat preparation (Figure 2B).

***Note:*** It is very important to add pre-chilled methanol and to shake the tube vigorously after adding methanol for proper devitillinization of the embryos. After devitillinization, the embryos should appear whitish in color as compared to non-devitellinized embryos which appear yellowish in color. Devitellinized embryo sink to the bottom of the tube, and embryos with vitelline membrane still attached, float at the surface or interface. Problem 3.

**CRITICAL:** Formaldehyde is toxic. Always use in a fume hood.

**Pause point:** These dechorionated, devitellinized and fixed embryos can be safely stored at −20°C in methanol and used for one month. This step is useful for pooling embryos of lines with low rate of oviposition.

### Dissection and fixation of larval tissue

**Timing: 1 h**

In *Drosophila,* the effects of a genetic manipulation may not manifest in the nuclei of early embryos because of masking due to maternal deposition of the molecule of interest. In such cases, the phenotype would be visible only at later stages of embryonic development. This necessitates to use the protocol in older embryos or specific tissues in the larval stages (salivary glands or imaginal disks). The next few steps detail the dissection and fixation of larval tissue for the purpose. The following steps (3–7) are not in continuation of steps 1 and 2, but run independently and converge with steps 1 and 2 at step 8.

3. Transfer the 3^rd^ instar larvae of desired phenotype with the help of a brush in a glass cavity block with 1× PBS. Wash the larvae of any adhering food material.
4. Dissect out the tissue of interest (salivary glands or imaginal discs) with the help of dissecting needles and forceps. Dissecting 20 larvae to yield 20 pairs of salivary glands/20 pairs of different imaginal discs is good enough for the purpose.
5. It is good to keep the glands/discs in bunches without separating them in individual entities, as this prevents their loss. They can be separated after the completion of the whole process, just before mounting on a slide for observation.
  a. Also place the dissected tissues in chambered slides. Usually, it is difficult to view dissected tissues in an Eppendorf tube and this results in loss of material during pipetting steps.
  b. Fixation, washings and all the processes for *in situ* NuMat preparation can be carried out in chambered slide, under the view of an inverted microscope. In such a situation, instead of using a rotating wheel, a platform shaker should be used for shaking during incubation.
  c. Addition or removal of solution should be done while keeping the tip of pipette tip in view and avoid sucking out of the tissue. This prevents the loss of any material during processing.
6. Remove the 1× PBS and replace it with fixation buffer and incubate it at 25°C for 20 min.
7. Remove the fixation buffer and proceed to steps for *in situ* NuMat preparation as detailed below.

### *In situ* NuMat preparation

**Timing: 3–4 h**

8. Allow embryos to settle down and discard methanol by gentle aspiration.
9. Wash the embryos by filling up the tube with 1 mL PBT (can be prepared and stored at 4°C for a month) and allowing the embryos to settle to the bottom.
10. Repeat for three times in quick succession. When using larval tissue, fixed tissue is also washed with PBT three times in chambered slides.
11. Rehydrate the embryos three times for 20 min each with 1 mL pre-chilled PBT with rotation at 40 rpm at 25°C. Such rehydration is not needed for tissues as they are already in aqueous medium.

***Note:*** Aliquot few embryos/tissues in a 1.5 mL of microfuge tube in 0.5 mL PBT and store at 4°C. Store two such aliquots if both immuno-fluorescence and TEM analysis are required. These aliquots serve as the controls which are not treated with salt extractions and DNase I digestion. These untreated control embryos/tissues should be processed for immuno-fluorescence and TEM simultaneously along with *in situ* NuMat embryos/tissues.

12. Add 1 mL PBT to the embryos/tissues in 1.5 mL microfuge tube and incubate the tube at 25°C for 20 min (Stabilization). This step can be omitted if stabilization is not deemed necessary. Discard supernatant by gentle aspiration.
13. Add 1 mL EB I (prepare and use on the same day) to the tube/chambered slide and incubate on rotating wheel/platform shaker at 40 rpm for 20 min at 25°C (salt extraction I).
14. Allow embryos/tissues to settle down and discard supernatant by gentle aspiration. Add fresh 1 mL EB II (prepare and use on the same day) and incubate at 40 rpm for 20 min (salt extraction II).
15. Wash embryos/tissues three times (10 min each) with PBT by adding the buffer and rotating/shaking the tube/chambered slide at 40 rpm.
16. Allow the tube to stand for 2 min to let the embryos/tissues settle at the bottom and remove the buffer by careful aspiration after the embryos/tissues settle down to the bottom of the tube.
17. Allow embryos/tissues to settle down and discard supernatant. Add 100 μL of DB containing 2μL of DNase I (10 mg/mL) (prepare and use on the same day) and incubate at 37°C shaker incubator for 50 min at 200 rpm.

***Note:*** The temperature should be maintained at 37°C for efficient DNA digestion. For insufficient digestion with DNase I, refer to Problem 4.

Improper fixation of samples may also result in under/over digestion and extraction of chromatin. In such a case, refer to Problem 5.

18. Repeat step 15 for washing. The settled embryos/tissues with *in situ* NuMat prepared are ready for observation.
19. Split embryos/tissues from 1.5 mL microfuge tube to two microfuge tubes and proceed for immuno-fluorescence or TEM observations.

### Immuno-staining of *in situ* NuMat preparation

One of the most convenient and informative ways to study the components of nuclear architecture is to visualize their localization and abundance by immuno-staining. The next few steps describe the protocol to immuno-stain an *in situ* NuMat protein component. Dilutions of primary and secondary antibodies used have been mentioned in the Key Resources Table in brackets in the first column

20. Dilute primary antibody according to empirically optimized dilution and add to untreated control embryos/tissues and *in situ* NuMat in 0.5 mL PBT, seal tubes with parafilm and incubate with rotation for 3 h at 25°C. For tissue, the primary antibody dilution can be added to the chambered slide and incubated.

***Note:*** Alternatively, the primary antibody incubation can also be carried out for 12–16 h at 4°C.

21. Repeat step 15 for washing.
22. Dilute secondary antibody according to empirically optimized dilution and add to untreated control embryos/tissues and *in situ* NuMat in 0.5 mL PBT, seal tubes with parafilm and incubate with rotation for 3 h at 25°C.

***Note:*** Secondary antibody is fluorescently labeled so all the steps following this till step 25 should be done in such a way that the sample is not exposed to direct light. For this, the tubes in which the secondary incubation is done should be well wrapped with aluminum foil to prevent exposure to light.

23. Repeat step 15 for washing.
24. After the last wash with PBT, allow the embryos/tissues to settle and discard supernatant with gentle aspiration. Transfer embryos/tissues on a glass slide by adding a small amount of PBT and pipetting the embryos/tissues along with the solution.

***Note:*** Use cut tips for pipetting out these embryos/tissues from the microfuge tubes and putting them on glass slides. Once the embryos/tissues have been transferred on the slide, discard excess solution from the slides with gentle aspiration without losing any material in the process.

25. Add mounting media along with DAPI in the required quantity.

**CRITICAL:** DAPI is potentially carcinogenic. Handle with care.

26. Gently place the coverslips with the help of a needle on top of the embryos/tissues preventing any air bubble to enter. Care should be taken not to squash the embryos/tissues. Seal the coverslip from sides by gently applying a transparent nail polish on all four sides of the cover slip.

***Note:*** These slides should be stored in boxes or slide book where they are not in contact with direct light.

**Pause point:** The slides can be scanned immediately or stored for 16 h at 4°C in slide boxes or slide books. However, these should be scanned within one day of preparation.

The slides can be scanned using a confocal or multiphoton microscope. Here we have used Leica SP8 microscope to image the slides. All the images were taken with 20× and 63× objectives. The acquired images were processed using LAS X software to generate the projection images.

### Processing of *in situ* NuMat preparation for TEM

Sometimes it might be desirable to look at the ultrastructure of the nuclear architecture at a higher resolution. This can be achieved by transmission electron microscopy imaging. The next few steps describe the same.

27. Fix untreated control embryos and *in situ* NuMat carrying embryos/tissues by adding 2% glutaraldehyde in 0.1 M cacodylate buffer and incubating samples with rotation for 1 h at 4°C.

**CRITICAL:** Glutaraldehyde is toxic. Handle with care in a fume hood.

28. Let the sample settle down under gravity and discard supernatant by gentle aspiration.
29. Wash the sample with 0.1 M cacodylate buffer three times by adding the cacodylate buffer, incubating with rotation for 5 min at 4°C, letting the sample to settle down under gravity and discarding the supernatant.

**Pause point:** The samples can be stored at 4°C for 12–16 h in 0.1 M cacodylate buffer after glutaraldehyde fixation.

30. Postfix samples with 1% OsO_4_ in 0.1 M cacodylate buffer and incubate with rotation for 30 min at 4°C.

**CRITICAL:** OsO_4_ is extremely toxic. Handle with care in a fume hood. A face mask with protection eyewear and protection for skin contact is essential.

31. Dehydrate samples with 35% ethanol in 0.1 M cacodylate buffer and incubate with rotation for 10 min at 4°C.
32. Allow samples to settle and discard supernatant.
33. Repeat steps 31 and 32 with following concentrations of ethanol: 50%, 70%, 80%, 90%, 95%, and 100%. Repeat the wash in 100% for one more time. Ethanol dilutions should be made fresh just before use.
34. Add ethanol:n-butyl alcohol (nBA) in 2:1 ratio and incubate with rotation at 25°C for 10 min. Ethanol:nBA dilutions should be made fresh just before use.
35. Allow samples to settle and discard supernatant by gentle aspiration. Add ethanol:nBA in 1:2 ratio and incubate with rotation at 25°C for 10 min.
36. Allow samples to settle down and discard supernatant by gentle aspiration. Add 100% n-BA and incubate with rotation at 25°C for 10 min.
37. Allow samples to settle down and discard supernatant. Add n-BA:DGD in the ratio 2:1 and incubate with rotation at 60°C for 1 h nBA:DGD dilutions should be made fresh just before use.
38. Allow samples to settle down and discard supernatant. Add n-BA:DGD in the ration 1:2 and incubate with rotation at 60°C for 1 h.
39. Allow samples to settle down and discard supernatant. Add 100% DGD and incubate with rotation for 1 h.
40. Repeat step 39.
41. Incubate samples along with the DGD at 25°C to allow it to solidify. If samples are stuck on the side of the tube bring them to the bottom by pipetting little amount of DGD on the sides of the tube.

**Pause point:** The DGD embedded samples can be stored indefinitely at 25°C.

42. Cut the microfuge tubes with a sharp blade and take out the DGD block containing sample.
43. Trim the sample and cut 70–90 nm thick sections using glass knife on an ultramicrotome at an angle of 10° and float the sections on water.
44. Transfer the sections on a formvar coated copper grids using a loop and handle and allow to dry for 2 h.
45. Remove embedding DGD by dipping the copper grids containing sections in n-BA and incubating for 12–16 h.
46. Dry grids. Observe and capture images using TEM at 120 V.

**CRITICAL:** Steps 42–46 should be done in dust free environment to avoid contamination of sections and EM grids.

## Expected outcomes

The protocol presented here is to isolate NuMat *in situ* in the whole embryo or dissected larval tissues. Most of the studies till now have been carried out either in cultured cells or isolated nuclei. Information gathered by such studies is limited and should be inferred with caution as they may disturb the nuclear architecture and genome organization and hence may not reflect the *in vivo* conditions faithfully. Our method has the potential to be a powerful means to define and analyze the components of nuclear architecture in a biologically relevant setup.

The utility of our method increases manifold as it can be used in conjunction with genetic experiments. The power of *Drosophila* genetics in the study of nuclear architecture has remained unharnessed because of the lack of a methodology to visualize NuMat in the context of the intact organism or tissue. Our method uses the developing embryo and larval tissues of *D. melanogaster* to prepare NuMat *in situ* and thus makes it possible to visualize nuclear architecture in the organism. *Drosophila* genetics combined with the presented method of *in situ* NuMat preparation provides a robust method to study various components of nuclear architecture in the background of the vast repertoire of mutations available in *Drosophila*.

**Figure 3:**
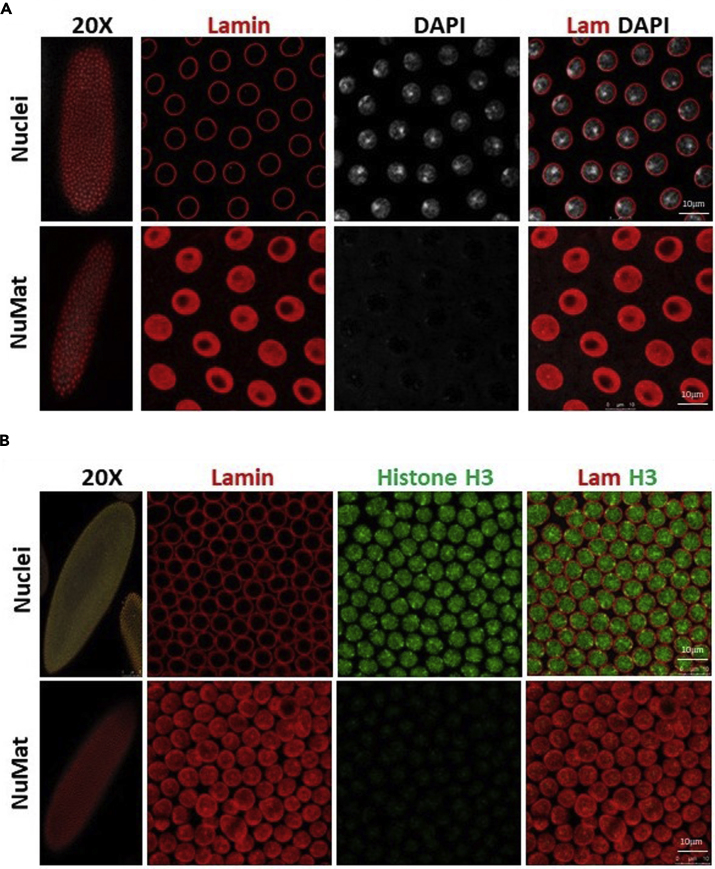
Visualization of *in situ* NuMat in early embryos *D. melanogaster* embryos at early stages of development (0–2 h) were used to prepare NuMat *in situ*. (A) Confocal images of unextracted embryo and embryo with *in situ* NuMat, immuno-stained with anti-Lamin Dm0 and DAPI. In unextracted embryos, Lamin Dm0 appears as a ring at the nuclear periphery of intact nuclei. After *in situ* NuMat preparation, no DAPI staining is observed in the nucleus, as chromatin has been digested and extracted out. Lamin Dm0 staining can now be seen in the nuclear interior as well. (B) Confocal images of unextracted embryo and embryo with *in situ* NuMat, immuno-stained with anti-His3 and anti-Lamin Dm0. Intact nuclei show prominent staining for histone H3 which is reduced to negligible in NuMat. The whole embryo images were taken with 20× objective and the higher magnification images were taken with 63× objective using Leica SP8 confocal microscope. Images were processed using LAS X software from Leica. Scale bar – 10 μm.

**Figure 4:**
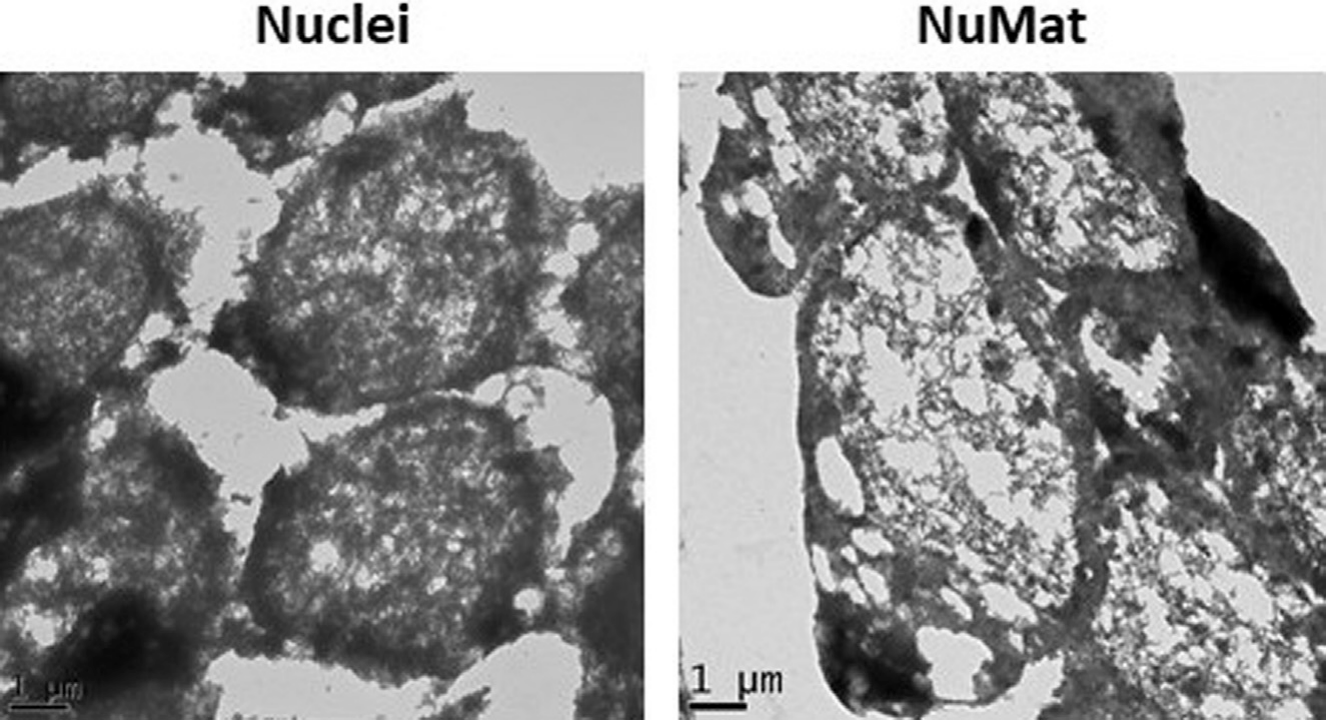
Visualization by TEM Images obtained by TEM of resinless sections of embryos carrying intact nuclei and *in situ* NuMat. Fine filaments are revealed in the NuMat after removal of chromatin. Scale bar – 1 μm.

**Figure 5:**
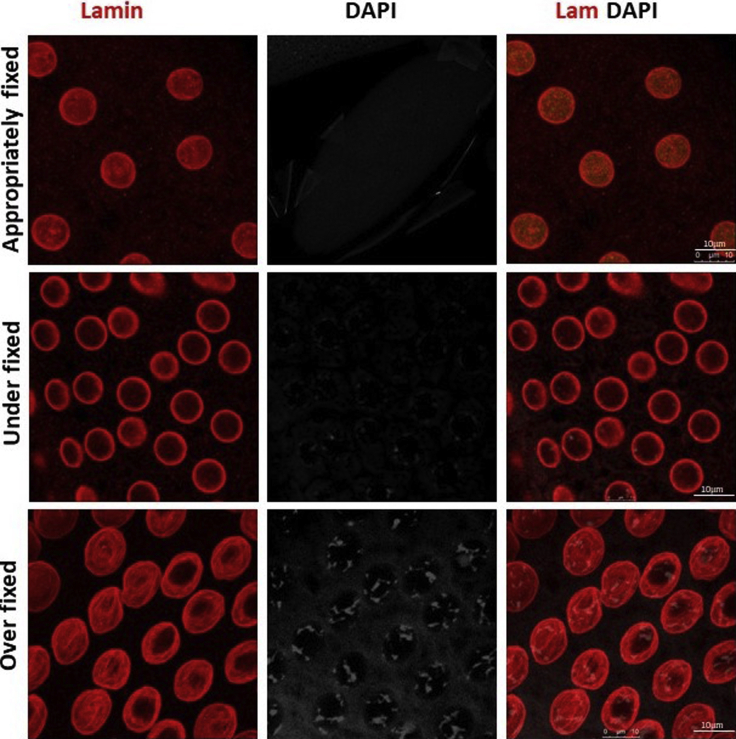
*In situ* NuMat prepared with under-fixed and over-fixed embryos A good *in situ* NuMat preparation requires appropriate fixation. As seen in the images in upper row, appropriate fixation results in near complete removal of chromatin as visualized by DAPI and intra-nuclear Lamin Dm0 staining. When the embryos are not sufficiently fixed or are not stabilized, the internal lamin staining is not visible (middle row). Over-crosslinking/stabilization results in clumps of DNA that remains unextracted as visualized by DAPI staining (lower row). Scale bar – 10 μm.

In this protocol the components of nuclear architecture are revealed by removing the bulk of dense chromatin by extraction with non-ionic detergent and salt followed by DNase I digestion. The NuMat thus revealed, consists of a nuclear lamina, an internal matrix composed of thick polymorphic fibers and nucleoprotein particles and remnants of nucleoli. The quality of nuclear matrices prepared is ascertained by visualization by confocal and TEM imaging (Figures 3 and 4). Complete removal of chromatin (no DAPI staining) indicates that NuMat is successfully prepared (Figure 3A). The same can also be assessed by immuno-staining the preparation with histone antibody where again near complete removal of histone is indicative of a good NuMat preparation (Figure 3B). However, at times, the fixation and stabilization steps can be over/under done and in such cases the *in situ* NuMat is not revealed efficiently. Internal lamin is not seen in improperly fixed and unstabilized NuMat preparation (Figure 5, compare upper panel with appropriate fixation to middle panel with under fixation). On the other hand, if the nuclei are over-fixed, it is difficult to extract the digested chromatin efficiently and we can see DAPI stained clumps of un-extracted chromatin that remains stuck in the NuMat (Figure 5, lower panel). Crosslinking and stabilization can be excluded altogether from the protocol, if the protein of interest is abundant and is sufficiently retained in NuMat even without these treatments. Protein of interest can be queried on this template of *in situ* NuMat by immuno-staining.

Figure 4 shows TEM image of intact nuclei and *in situ* NuMat preparation. The dense chromatin granules are removed in the *in situ* NuMat preparation revealing the extraction resistant fibro-granular meshwork. Such imaging is helpful in understanding the ultra-structure of nucleus at highest resolution.

**Figure 6:**
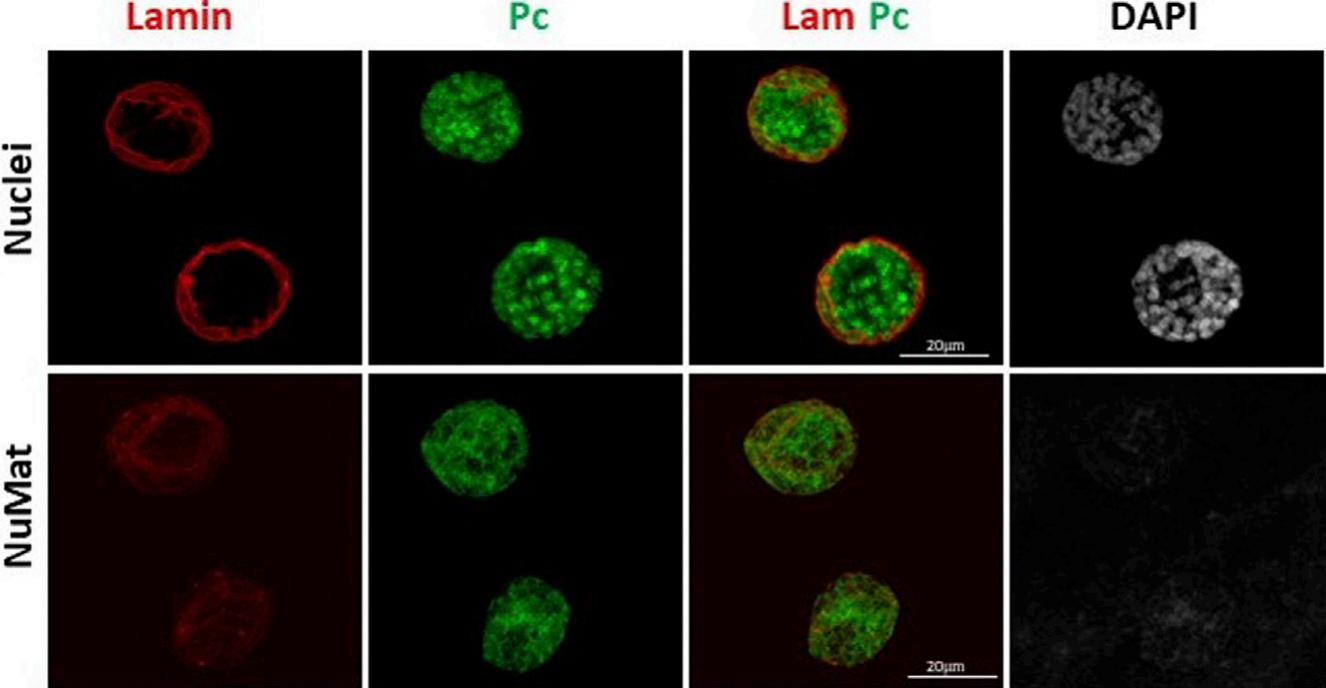
Visualization of *in situ* NuMat in salivary gland nuclei *In situ* NuMat prepared with *D. melanogaster* 3^rd^ instar larval salivary gland shows that the bulk of chromatin present in polytene chromosomes is efficiently extracted (assessed by loss of DAPI staining) to reveal the nuclear architecture of salivary gland nucleus. Immuno-staining with anti-Pc (Polycomb) shows the association of this protein with nuclear architecture. Scale bar – 20 μm.

The strength of *in situ* protocol lies in the proposition that the power of *Drosophila* genetics can be harnessed to uncover novel molecular players with a role in nuclear architecture. However, in *Drosophila,* the effects of a genetic manipulation may not manifest in the nuclei of early embryos because of masking due to maternal deposition of the molecule of interest in the embryo. In such cases, the phenotype would be visible only at later stages of embryonic development. This necessitates that the protocol works effectively in older embryos or specific tissues in the larval or adult stages. As seen in Figure 6, the chromatin digestion and salt extraction, works well in larval salivary glands. Negligible staining with DAPI indicates efficient removal of chromatin and Lamin Dm0 in the nuclear interior is revealed defining the NuMat. As an example, we have stained the protein ‘Polycomb’ for its localization in nuclear architecture. In Figure 6 we can see that the protein binds to polytene chromosome bands in intact nuclei. After *in situ* NuMat preparation, chromatin is removed as assessed by DAPI staining. The polytene nuclei lose its shape and intra-nuclear lamin is revealed. Interestingly ample amount of Pc protein is retained in the NuMat hinting an architectural association of PcG silencing complexes.

With these examples we show that the *in situ* NuMat isolation protocol can prove to be a robust method to study spatial epigenetics where nuclear structure and function are linked. Moreover, as NuMat is cell type specific and serves to define chromosome territories, studying it in context of whole organism would be highly informative (Fey and Penman, 1988; Ma et al., 1999). NuMat also serves as a platform most of the nuclear functions (Berezney and Coffey, 1975; McCready and Cook, 1984; Jackson and Cook, 1985; Zeitlin et al., 1987) and thus the protocol presented is a powerful means to define nuclear macromolecular complexes and study their function in architectural context.

## Limitations

As this technique uses whole embryos/tissue without the isolation of pure nuclei, our protocol may not be suitable for biochemical experiments such as westerns, proteomics, etc.

## Troubleshooting

### Problem 1

No immuno-staining with antibodies.

### Potential solution

Apart from the common problem of proper antibody dilution etc., one of the main reasons for this could be that the very first step of dechorionation of embryos was not carried out properly. It is essential that the removal of chorion is monitored under a stereomicroscope and optimized. (step 1).

### Problem 2

Embryos do not segregate properly on heptane-PBS interface.

### Potential solution

This results from insufficient rinsing and incomplete removal of bleach. Instead of forming a clearly discernible ring, embryos are seen dispersed in a mixture of heptane and PBS. (step 2).

### Problem 3

Extra material still attached to the embryo, attracting non-specific staining.

### Potential solution

This results from picking embryos that are not segregated in the specified manner at heptane – PBS step and heptane – methanol step. Embryos of good quality settle down promptly and other embryos floating about should not be included in the preparation. Including these embryos will also cause clumping when thawing embryos stored in cold methanol. (step 2).

### Problem 4

Improper digestion with DNase I.

### Potential solution

One of the reasons can be that the bleach was not washed out properly and remains to interfere with the enzymatic digestion. After dechorionation, wash bleach thoroughly with running water.

Optimize the concentration and timing of DNase I digestion for complete removal of chromatin as assessed by DAPI staining. (step 17).

### Problem 5

Clumps of unextracted chromatin remain in the NuMat.

### Potential solution

This results from over-fixation and over-stabilization of samples. Without altering the concentration of formaldehyde (4%) or temperature of fixation/stabilization (25°C), optimize the timing of fixation/stabilization and it can be reduced from 20 min to 10 min as needed. (step 17).

## Resource availability

### Lead contact

Further information and requests for resources and reagents should be directed to and will be fulfilled by the lead contact, Rakesh K Mishra (mishra@ccmb.res.in).

### Materials availability

This study did not generate any unique reagents. All reagents are available as specified in key resources table.

## Data Availability

This study did not generate datasets/codes.
